# Evaluation of Dietary Administration of Chestnut and Quebracho Tannins on Growth, Serum Metabolites and Fecal Parameters of Weaned Piglets

**DOI:** 10.3390/ani10111945

**Published:** 2020-10-22

**Authors:** Valentina Caprarulo, Monika Hejna, Carlotta Giromini, Yanhong Liu, Matteo Dell’Anno, Stefania Sotira, Serena Reggi, Carlo Angelo Sgoifo-Rossi, Maria Luisa Callegari, Luciana Rossi

**Affiliations:** 1 Department of Health, Animal Science and Food Safety, University of Milan, 20133 Milan, Italy; valentina.caprarulo@unimi.it (V.C.); carlotta.giromini@unimi.it (C.G.); matteo.dellanno@unimi.it (M.D.); stefania.sotira@unimi.it (S.S.); serena.reggi@unimi.it (S.R.); carlo.sgoifo@unimi.it (C.A.S.-R.); luciana.rossi@unimi.it (L.R.); 2 Department of Animal Science, University of California, Davis, CA 95616, USA; yahliu@ucdavis.edu; 3 Department of sustainable food process, Faculty of Agriculture, Food and Environmental Sciences, Università Cattolica del Sacro Cuore, via Emilia Parmense 84, 29122 Piacenza, Italy; marialuisa.callegari@unicatt.it

**Keywords:** tannins, plant extracts, weaned piglets, zootechnical performances, blood metabolites, fecal nitrogen concentration

## Abstract

**Simple Summary:**

In pig livestock, alternatives to in-feed antibiotics are needed to promote health status and to control enteric infections. Plant extracts containing biologically-active molecules may influence animal metabolism and performance, therefore potentially reducing the use of antibiotics in pigs. Tannins have antioxidant, anti-inflammatory and antimicrobial properties, and have been adopted to enhance growth performance, modulate intestinal microbiota, and decrease the incidence of diarrhea, particularly during the post-weaning period. Despite their functional properties, tannins are known to be astringent compounds due to their ability to complex and precipitate proteins, particularly proline-rich proteins. The common use of non-standardized plant extracts generates results that are often controversial and difficult to interpret. In this study, attention was focused on the evaluation of the inclusion of a mixture of tannins extracted from chestnut and quebracho in the diet of piglets.

**Abstract:**

In pig livestock, alternatives to in-feed antibiotics are needed to control enteric infections. Plant extracts such as tannins can represent an alternative as a natural source of functional compounds. The aim of this study was to evaluate the in vitro digestibility and in vivo effects of oral supplementation of combined chestnut (Ch) and quebracho (Qu) tannins in order to establish if they can induce a positive effect on weaned piglets’ performance, metabolic status and fecal parameters. In vitro digestibility (dry matter, DM) of diets was calculated using a multi-step enzymatic technique. In vitro digested diet samples were further tested on an intestinal porcine enterocyte cell line (IPEC-J2). Weaned piglets (*n* = 120; 28 ± 2 day old) were randomly allotted to two groups (12 pens in total with 10 pigs per pen): control (Ctrl) and treatment (Ch/Qu). After one week of adaptation (day 0), 35-day-old piglets in the Ctrl group were fed a Ctrl diet and the Ch/Qu group were fed with 1.25% Ch/Qu for 40 days. Body weight and feed intake per pen were recorded weekly. At day 40, blood and fecal samples were collected. Principal metabolic parameters were evaluated from blood samples by enzymatic colorimetric analysis. Total phenolic compounds, urea, and ammonia in feces were analyzed (Megazyme International, Bray, Ireland). In vitro digestibility and cell viability assays showed that the inclusion of 1.25% Ch/Qu slightly reduced diet digestibility compared with the Ctrl diet, while intestinal cell viability was not altered with low concentrations of Ch/Qu digesta compared with Ctrl. In vivo results did not show any adverse effects of Ch/Qu on feed intake and growth performance, confirming that dietary inclusion of Ch/Qu at a concentration of 1.25% did not impair animal performance. The decreased diet DM digestibility in the Ch/Qu diet may cause increased serum concentration of albumin (Ctrl: 19.30 ± 0.88; Ch/Qu: 23.05 ± 0.88) and albumin/globulin ratio (Ctrl: 0.58 ± 0.04; Ch/Qu: 0.82 ± 0.04), but decreased creatinine (Ctrl: 78.92 ± 4.18; Ch/Qu: 54.82 ± 4.18) and urea (Ctrl: 2.18 ± 0.19; Ch/Qu: 0.95 ± 0.19) compared with Ctrl. Pigs in the Ch/Qu group contained higher (*p* < 0.05) concentrations of fecal phenolic compounds and nitrogen than the Ctrl group, while fecal ammonia and urea were not affected by tannins. In conclusion, Ch/Qu tannin supplementation did not influence growth performance. Although lower digestibility was observed in the diet supplemented with Ch/Qu tannins, Ch/Qu supplementation did not show any adverse effect on intestinal epithelial cell viability.

## 1. Introduction

In swine production, weaning is recognized as the most critical phase because piglets are exposed to various biological stressors, including physiological, environmental, and social changes, which lead to increases in the exposure to pathogens and dietary or environmental antigens [1,2,3]. For these reasons, antibiotics are often used to control bacterial infections and post-weaning diarrhea.

Livestock antibiotic resistance is a major global threat of increasing concern for animal and human health, which has thus led the European Union to ban the use of antibiotics as growth-promoting agents [4,5]. As a result, in the last decade, an increased use of zinc oxide at a pharmacological level (ZnO, 2000–3000 ppm) was observed as an alternative to antibiotics [6]. However, the widespread use of pharmacological levels of ZnO has raised concerns related to environmental issues and the potential increase in the prevalence of antibiotic resistant bacteria [4,7,8,9]. The Agency’s Committee for Medicinal Products for Veterinary Use (CVMP) recommended the withdrawal of the existing marketing authorizations for veterinary medicinal products containing zinc oxide (EMA/394961/2017) [10].

Due to such restrictions, alternatives to antibiotics and alternatives to zinc oxide are urgently needed to guarantee animal production in line with health principles [11,12]. In this scenario, plant extracts can represent a valuable alternative as a natural source of functional compounds, such as polyphenolic compounds [13,14,15,16,17]. Among polyphenols, tannins derived from plant extracts are the largest class which can be classified into hydrolysable (HTs) or condensed (CTs) subgroups [18]. Tannins have been tested in the poultry and swine sectors, initially as tannin-rich feedstuffs (such as sorghum, barley, maize and fava beans), and more recently as tannin extracts from different plants (grape seed, grape pomace, acorns, oak, green tea leaves, and pomegranate) [19,20]. Chestnut trees (Ch, *Castanea sativa* Mill.) are a source of HTs, whereas quebracho trees (Qu, *Schinopsis* spp.) are a source of CTs or proanthocyanidins [21]. Tannins extracted from these plants have been applied in intensive swine farms due to their antioxidant, anti-inflammatory, and antibacterial activities [16,21].

Heterogeneous results were obtained on the effects of Ch and Qu to enhance growth performance, modulate intestinal microbiota, and decrease the incidence of diarrhea during the post-weaning period [22,23,24]. These heterogeneous results could be related to the chemical characteristics of tannins, which can compromise the palatability, digestibility, and protein use of feed. The ability to bind proteins and carbohydrates in monogastric animals is associated with the antinutritional effects of tannins in reducing feed palatability [18]. Thus, contrasting results on the effective supplementation of tannins on pigs’ performance and intestinal health have been observed in relation to the source of tannins (Ch and Qu), dosage of tannins, and the type of tannins (HTs or CTs) included in the diets [25,26]. Therefore, the heterogeneity of commercial products is associated with the use of Ch or Qu individually or in mixtures with different percentages of tannins (from 54% to 82%) and hence different amounts of HTs and CTs.

However, the studies presented in the literature for pigs in which Ch and Qu has been used showed that most of the products mainly contained tannins extracted from Ch (a source of HTs) [22,25,26]; no studies that adopted the use of Qu tannins (a source of CTs) have been reported and only a few studies that adopted the combination of Ch and Qu tannins (source of HTs/CTs) have been published [26,27,28,29]. Further research to fully understand the synergistic effect of HTs/CTs, derived from both chestnut and quebracho, on the growth performance of weaned piglets is needed.

In light of this, identifying the correct application dose is essential in order to maximize the beneficial effects of tannins, and minimize the antinutritional effects on animal growth performance and health. Thus, the main purpose of this study was to evaluate the in vitro dry matter (DM) digestibility and in vivo effect of Ch/Qu in order to establish if the inclusion of 1.25% combined chestnut and quebracho tannins can induce a positive effect on weaned piglets.

## 2. Materials and Methods

### 2.1. Animals, Housing, Experimental Design and Treatment

Our in vivo trial complied with Italian regulations on animal experimentation and ethics (DL 26/2014) [30] in accordance with European regulation (Dir. 2010/6) [31] and was approved by the Animal Welfare Body of the University of Milan (number 31/2019). This trial was performed in an intensive conventional herd farm located in Lombardy (Italy) that was free from any of the diseases reported in the previous A-list of the International Office of Epizootics, and free from Aujeszky’s disease, atrophic rhinitis, transmissible gastroenteritis, porcine reproductive and respiratory syndrome, and salmonellosis.

A total of 120 crossbred piglets (Large White × Landrace), weaned at 28 ± 2 days (50% female and 50% male), were identified using individual ear tags and allotted in randomized complete block design into two experimental groups: control group (Ctrl) and treatment group (Ch/Qu). There were 60 pigs per treatment with 6 replicate pens and 10 pigs per pen. The groups were homogeneous in terms of gender, weight and litter. After one week of adaptation (considered day 0, piglets were 35 days old), during which all animals received the same basal diet, the experimental diets were distributed ad libitum to all animals for 40 days. Experimental diets (Plurimix, Fabermatica, CR, Italy) were formulated according to animal requirements for the post-weaning phase (Ferraroni Mangimi SpA, Bonemerse, Italy). The two diets were isoenergetic and isoproteic and fulfilled the NRC (2012) [32] requirements for post-weaned piglets (Table 1). The Ch/Qu diet was differentiable by the inclusion of 1.25% of tannin extract from chestnut and quebracho trees (Silvafeed for Swine, Silvateam, Italy).

The enrolled piglets of both experimental groups were reared in one unique room at constant temperature (27 °C) and humidity (60%) for the entire experimental period. The room had an unobstructed floor area available to each weaner piglet of 0.40 m^2^, according to Directive 2008/120/EC [33]. Each pen was equipped with nipple drinkers with ad libitum access to fresh water.

### 2.2. Chemical Analyses and In Vitro Digestibility Evaluation of Ctrl and Ch/Qu Diets

Feed samples from Ctrl and Ch/Qu (500 g each) were collected in order to guarantee the representativeness of samples according to the Reg. 152/2009/EC [34] and ISO 24333:2009 [35]. Moreover, a total of 50 g of commercial product supplemented in the Ch/Qu diet (Silvafeed Nutri P/ENC for Swine, Silvateam, Italy) was collected. The Ctrl and Ch/Qu diets as well as the tannin supplements were analyzed for proximate analysis, including moisture, crude protein (CP), crude fibre (CF), ether extract (EE), and crude ash [36]. Specifically, moisture determination was performed by oven-drying at 135 °C for 2 h. Crude protein content was measured according to the Kjeldahl method. Crude fiber was determined by the Filter Bag technique. Ether extract content was determined by the Soxhlet method with prior hydrolysis. Ash was measured using a muffle furnace at 550 °C. Fecal samples were also analyzed for moisture following the procedure described above.

Total phenolic compounds in Ctrl and Ch/Qu diets and in Ch/Qu tannin extracts were assayed according to the Folin-Ciocalteu method [37]. Each feed sample was weighed (5 ± 0.5 g) and mixed with 30 mL of pure methanol (Sigma Chemical Co, St. Louis, MO, USA) for 24 h at room temperature and subsequently filtered (Whatman 54, Florham Park, NJ, USA). The obtained chemical extracts from feed samples were tested for total phenolic compounds. Prior to the analysis, a standard curve was prepared using tannic acid (Sigma Chemical Co, St. Louis, MO, USA). The tannic acid was water-dissolved to obtain a stock solution of 960 μg/mL. Dilutions of the stock solution were prepared to obtain final concentrations from 60 to 960 μg tannic acid/mL. The Folin-Ciocalteu reagent was diluted 1:10 with deionized water and a solution of 1 M sodium carbonate (Sigma Chemical Co, St. Louis, MO, USA) was prepared. Briefly, an aliquot (0.5 mL) of extract, blank or standard was placed in a 15 mL plastic tube, where the Folin-Ciocalteu reagent (2.5 mL) and sodium carbonate (2 mL) were added and the mixture was incubated at room temperature in a dark chamber for 20 min. The total phenolic content was determined by colorimetry at 630 nm using an UV-visible spectrophotometer (Jasco V-630, Easton, MD, USA). Total phenolic content was expressed as tannic acid equivalents (g TAE/kg). The analyses were performed in technical duplicate and biological triplicate.

The Ctrl and Ch/Qu diets adopted in the in vivo trial were in vitro digested using the protocol reported by Reggi et al. [16]. The in vitro digestion was performed according to the protocol described by Regmi et al. [38] with minor adaptations previously reported by our group [39]. At the end of the in vitro digestion procedure, a soluble fraction and an undigested fraction (UF) were obtained. The soluble fraction was used for cell viability assays (detailed below). The UF was then collected in a filtration unit using a porcelain filtration funnel lined with pre-weighed filter paper (Whatman no. 54). The UF, along with the filter paper, were dried overnight at 65 °C. The UF was used to calculate the in vitro digestibility (IVD) using Equation (1):

IVD (%) = (sample (DM) − sample UF (DM))/(sample (DM) × 100).(1)

The digestion procedure was performed twice (2 biological replicates). Whey protein (90%) was included as a reference sample for stability tests in all digestions performed, as previously indicated in Giromini et al. [17].

### 2.3. Effect of Ctrl and Ch/Qu Diet on Swine Intestinal Cell Viability

The intestinal porcine enterocyte cell line IPEC-J2 is unique as it is derived from the small intestine isolated from the jejunum of a neonatal unsuckled piglet (ACC 701, DSMZ, Braunschweig, Germany) and is not transformed nor tumorigenic in nature [39]. IPEC-J2 cells were cultured in Dulbecco’s Modified Eagle Medium + Ham’s F-12 mixture (DMEM/F-12) supplemented with HEPES (N-(2-Hydroxyethyl)piperazine-N′-(2-ethanesulfonic acid)), fetal bovine serum (FBS), penicillin/ streptomycin and cultivated in a humid chamber at 37 °C with 5% CO_2_. All experiments were performed using IPEC-J2 cells within six cell passages (passages 16 to 22) to ensure reproducibility. In particular, IPEC-J2 cells were seeded at a density of 1.5–2 × 10^5^ cells/mL in 96-well plates and cultured for 24 h.

Samples of in vitro digested Ctrl and Ch/Qu diets (soluble fraction of the in vitro digestion described above) were used to obtain a dose-response curve in IPEC-J2 cells. Diluted concentrations of digesta were applied to cells (21.31–0.33 mg/mL), while DMEM/F-12 mix alone was used as a negative control (0 mg/mL, DMEM). Cell viability was determined after a three-hour incubation by a colorimetric proliferation assay (the 3-(4,5-dimethylthiazol-2-yl)-2,5 diphenyltetrazolium bromide MTT test) in accordance with the manufacturer’s instructions (Sigma Chemical Co, St. Louis, MO, USA).

### 2.4. Collection of Fecal and Blood Samples

Fecal (*n* = 6) and serum blood samples (*n* = 12) were collected at day 40 of the in vivo trial (from 75-day-old piglets), according to ethical authorization, from a randomly selected subset of animals (blood: *n* = 12/treatment fecal: *n* = 6/treatment, 50% female and 50% male) for each treatment group cohort one hour before the morning feeding and within one hour after feeding in order to have homogeneous conditions and representative parameters. Fecal samples were individually collected from rectal ampulla and immediately stored at −20 °C until further analysis. From each piglet included in the subset, blood was collected from the jugular vein into vacuum tubes, maintained for 2 h at room temperature, and then centrifuged at 850 r.c.f. (relative centrifugal force) for 10 min at 4 °C. Serum was aliquot and stored at −20 °C for further analysis.

### 2.5. Zootechnical Evaluation

Piglets were individually weighed (BW) on day 0, 14, 28 and 40 of the in vivo trial (35-, 49-, 63- and 75-day-old piglets, respectively). The amount of feed offered ad libitum to the experimental groups (Ctrl and Ch/Qu) was recorded. The feed intake of individual pen (experimental unit for the feed intake evaluation) was calculated every week by measuring the total refusals. The average daily feed intake (ADFI) and feed-to-gain ratio were calculated from day 0 to 14, from day 14 to 28 and from day 28 to 40 of the in vivo trial (35–49-, 49–63- and 63–75-day-old piglets, respectively). Based on the ADFI and total phenolic compounds in the feed, phenolic compound intake was calculated from day 0 to 14, from day 14 to 28 and from day 28 to 40 of in vivo trial (35–49-, 49–63- and 63–75-day-old piglets, respectively). The health status of the piglets was monitored daily. Mortality was registered, and the incidence of diarrhea was calculated based on the number of piglets with clinical sign of diarrhea [11].

### 2.6. Total Phenolic Compounds, Urea and Ammonia in Feces

The total phenolic compounds in feces were evaluated as previously described. Fecal samples were weighed (5 ± 0.5 g) and mixed with 30 mL of pure methanol (Sigma Chemical Co, St. Louis, MO, USA) for 24 h at room temperature and subsequently filtered (Whatman 54, Florham Park, NJ, USA). The obtained chemical extracts from fecal samples were tested for total phenolic compounds according to the Folin-Ciocalteu method [37].

Fecal samples (5 g) were treated prior to analysis with 20 mL of perchloric acid (1 M). Then, samples were homogenized for 2 min using an Ultra-turrax (T25, Ika Works Inc., Wilmington, NC, USA). The homogenized samples were adjusted to pH 8 with KOH (2 M) and adjusted to the mark with 100 mL of distilled water. The samples were then maintained on ice for 20 min and centrifuged at 13,000× *g* for 10 min. The supernatant was filtered (Whatman 1, Florham Park, NJ, USA). A K-URAMR test kit (Megazyme, Bray, Ireland) was used for urea and ammonia analysis. The test kit method involved urease, which catalyzed the hydrolysis of urea to ammonia and the subsequent reaction of ammonia, 2-oxoglutarate and reduced nicotinamide-adenine dinucleotide phosphate (NADPH) in the presence of glutamate dehydrogenase to form glutamic acid and NADP+. The consumption of NADPH was measured by the decrease in absorbance at 340 nm using a UV-visible spectrophotometer (Jasco V-630, Easton, MD, USA) and was proportional to the original amount of urea over a finite range (Urea/Ammonia (Rapid) Assay Procedure K-URAMR 11/05, Megazyme International, Bray, Ireland). The analyses were performed in technical duplicate and biological triplicate.

### 2.7. Blood Serum Analysis

Serum biochemical analyses were performed by the Lombardy and Emilia Romagna Experimental Zootechnic Institute (IZSLER). The concentration of total protein (g/L), albumin (g/L), globulin (g/L), albumin/globulin (A/G ratio), alanine aminotransferase (ALT-GPT; IU/L), aspartate aminotransferase (AST-GOT; IU/L), alkaline phosphatase (ALP; IU/L), glucose (mmol/L), urea (mmol/L), creatinine (μmol/L), total bilirubin (umol/L), total cholesterol (mmol/L), triglycerides (mmol/L), high-density lipoprotein (HDL; mmol/L), low-density lipoprotein (LDL; mmol/L), calcium (mmol/L), phosphorus (mmol/L), and magnesium (mmol/L) were measured. The parameters were analyzed at 37 °C via standard enzymatic colorimetric analysis using a multiparametric autoanalyzer for clinical chemistry (ILab 650; Instrumentation Laboratory Company, Lexington, MA, USA).

### 2.8. Statistical Analysis

One-way ANOVA was calculated using SAS 9.4 (SAS Inst. Inc., Cary, NC, USA) and was used to analyze digestibility and cell viability data. Animal performance, phenolic compounds (ingestion and diet, supplement and fecal content), fecal protein, nitrogen, ammonia, urea and blood metabolite data were analyzed using a generalized linear mixed model through generalized linear mixed model Proc GLIMMIX SAS 9.4 (SAS Inst. Inc., Cary, NC, USA) [40]. For animal performance, the model included the fixed effect of treatments (Trt), experimental day (Day) and the interaction between the two factors (Trt × Day), and the repeated measures over time were included in the RANDOM statement. Tukey-Kramer studentized adjustments were used to separate treatment means within the two-way interactions. Within significant two-way interactions, the slice option was used to separate means within specific treatments and experimental days. The Proc CORR procedure was used to calculate and test Spearman correlations by treatment among feed (ADFI, phenolic compound intake), fecal (fecal phenolic compounds, protein, nitrogen, ammonia, urea) and blood metabolites (glucose, urea, total cholesterol, HDL, LDL, triglycerides) on day 40. Means were considered different when *p* ≤ 0.05. Results are reported as least squares means (LSMEANS) and standard errors of the means (SEM).

## 3. Results

### 3.1. Chemical Analyses and In Vitro Digestibility Evaluation of Experimental Ch/Qu and Ctrl Diets

The experimental diets contained a similar content of principal nutrients (Table 2). The phenolic content differed due to the tannin supplementation, and was 3.68 times higher in Ch/Qu group than in the Ctrl group. The in vitro experiments showed a slight reduction of DM digestibility in the Ch/Qu diet (69.33% of DM) compared to the Ctrl diet (72.00% of DM).

### 3.2. Swine Intestinal Cell Viability

**Figure 1:**
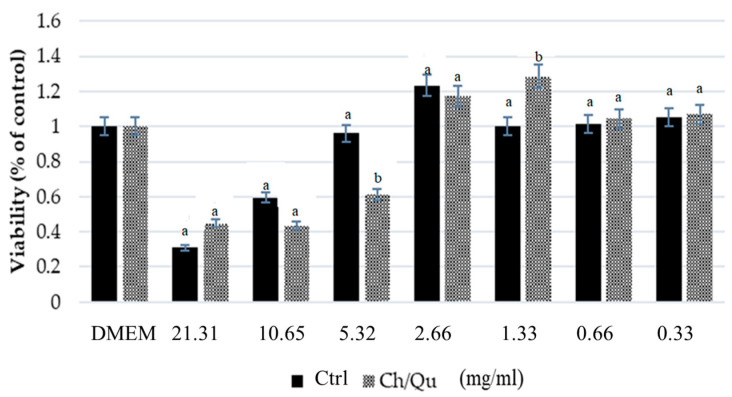
Effect of different concentrations (21.31–0.33 mg/mL) of Ctrl and Ch/Qu digesta (<3 kDa) on intestinal porcine epithelial cell line IPEC-J2 cell viability (via 3-(4,5-dimethylthiazol-2-yl)-2,5 diphenyltetrazolium bromide MTT assay). Data are expressed as percentage of control (DMEM - Dulbecco’s Modified Eagle Medium + Ham’s F-12 mixture, 0 mg/mL) as least squares means (LSMEANS) and standard errors of the means (SEM) (*n* = 3). At each concentration, different letters (a,b) denote statistical differences between Ch/Qu and Ctrl.

Samples of in vitro digested Ch/Qu and control diets were tested on IPEC-J2 cell viability at diluted concentrations. In general, Ch/Qu and Ctrl digesta showed comparable effects on cell viability at the tested concentrations. An exception was represented by the concentration of 5.32 mg/mL of digesta. At this concentration, Ch/Qu seems to detrimentally affect viability compared to Ctrl. In contrast, at a concentration of 1.33 mg/mL, Ch/Qu promoted cell viability compared to Ctrl (*p* < 0.05) (Figure 1).

### 3.3. Zootechnical Performance

No mortality and no veterinary interventions were observed in the Ch/Qu and Ctrl groups during the entire experimental period. Moreover, no differences between males and females were observed in the analyzed parameters. Tannin supplementation did not affect ADFI, BW, ADG or feed-to-gain ratio (Table 3). Daily phenolic compound intake was significantly higher (*p* < 0.01) in the Ch/Qu group compared to the Ctrl group during the entire experimental period.

Regarding the health status of the animals throughout the experimental period in both groups, clinical signs of diarrhea occurred after seven days and the signs were transient (on average lasting for three days). The occurrence of diarrhea (number of new animals with signs of diarrhea per group) was 3.39% and 5.00% in the Ctrl and Ch/Qu groups at day 0-14, respectively; at day 14–28, the incidence was 18.64% and 15.52% in the Ctrl and Ch/Qu groups; and at day 28–40, the incidence was 1.69% and 3.45% in the Ctrl and Ch/Qu groups (Supplementary Figure S1).

### 3.4. Blood Serum Metabolites

Dietary treatment with tannins did not influence total protein content, globulin, ALT-GPT, AST-GOT, ALP, glucose, total bilirubin, total cholesterol, triglycerides, HDL, LDL, calcium, phosphorus or magnesium (Table 4). Pigs in the Ch/Qu group had higher albumin (*p* = 0.006) and A/G ratio (*p* = 0.001), but lower creatinine (*p* < 0.001) and urea (*p* = 0.001) compared with pigs in the Ctrl group.

### 3.5. Influence of Dietary Treatment with Tannins on Feces

Weaned piglets in the Ch/Qu group showed higher concentrations of fecal phenolic compounds than the Ctrl group (*p* = 0.047). Fecal nitrogen concentrations were significantly higher (*p* = 0.002) in the Ch/Qu group than the Ctrl group, while fecal ammonia (*p* = 0.684) and urea (*p* = 0. 235) were not affected by the dietary inclusion of tannins (Table 5).

### 3.6. Influence of Dietary Treatment with Tannins on Correlation among Feed, Feces and Blood Parameters

We found a significantly positive correlation between fecal nitrogen and phenolic compound intake (*p* = 0.007). There was also a negative correlation between fecal nitrogen and blood urea (Table 6).

## 4. Discussion

The present study included two experimental sections. Firstly, an in vitro characterization of the Ch/Qu and Ctrl diets (chemical composition, in vitro digestibility (DM) and cell viability assays) was performed. Secondly, an in vivo experimental trial in swine was conducted during which the Ch/Qu and Ctrl diets were administered to animals.

The in vitro results showed that Ch/Qu in vitro digestibility was slightly but not significantly reduced compared with Ctrl diet digestibility, suggesting that the presence of Ch and Qu tannins in the diet may limit nutrient digestibility due to binding and forming stable and insoluble complexes with proteins [44,45].

In addition, results obtained in IPEC-J2 cells showed that the Ch/Qu diet had a similar effect on intestinal epithelial cell viability compared to the Ctrl diet. The tested concentration range allowed us to observe a hormetic effect in IPEC-J2 cell viability, as indicated by the fact that low concentrations were stimulatory while the high concentrations were harmful to cell viability [46]. Despite their antimicrobial and antioxidant properties, the supplementation of tannins in animal feed could decrease feed palatability and the absorption, digestion, and utilization of dietary proteins [18,47]. However, in our study, the feed intake was the same for the Ctrl and Ch/Qu groups; thus, the inclusion of 1.25% Ch/Qu tannins in the diet did not affect feed palatability. Moreover, growth performance of the Ctrl and Ch/Q groups did not show any significantly differences. In fact, BW and ADG remained at a similar level, confirming that the dietary inclusion of Ch/Qu did not impair animal performance due to the protein binding property of tannins [48].

Different literature cases have shown contradictory results relating to the dosages of tannin inclusion. In line with our study, the inclusion of 1%, 2% and 3% of Ch/Qu or Ch tannins had no effect on ADG, BW and feed efficiency in pigs [26,28], whereas Ch/Qu tannin supplementation at 2% showed a positive effect on ADFI and ADG [23]. Furthermore, Bee et al. [47] reported that the inclusion of 3% Ch/Qu tannins significantly decreased the gain-to-feed ratio in boars, while BW and ADG were not influenced. Moreover, lower doses (from 0.11% to 0.45%) of Ch tannins did not improve growth performance in piglets [22,27]. The contradictory literature results related to dosages could thus be related to different compositions of tannin-based commercial products. In light of this, the dosages of tannins cannot be a unique explanation for the effect on animal performance.

In fact, the combined effect of Ch/Qu tannins could be exacerbated during stressful conditions, such as experimental bacterial infections [24,28]. According to Reggi et al. [16], beneficial effects were reported when Ch/Qu digesta were administered to experimentally stressed intestinal swine cells, suggesting that it might have a trophic effect at the intestinal epithelium, and an increased viability of cells was observed after tannin treatment. In present study, animals were reared in a conventional herd farm. The incidence of diarrhea was not different between the Ctrl and Ch/Qu groups during the entire experimental period. Moreover, we observed a decrease of diarrhea incidence in the Qu/Ch group from day 14 to 28 when compared to the Ctrl group, and the occurrence of diarrhea was below the average post-weaning level [49,50].

The physiology and hematological parameters of animals can be influenced by several factors, including nutrition [51]. In the present study, all biochemical parameters for both the Ctrl and Ch/Qu groups were within the reference range of weaned pigs [41,42,43]. The increase in serum albumin and A/G ratio in the Ch/Qu group compared to the Ctrl group were in line with results by Chedea et al. [52]. Albumin is an important indicator of protein status; the increment of this serum metabolite could be due to increased microbial protein in the intestine, which might trigger and increase the amount of amino acids [53]. The level of the blood A/G ratio could be related to protein synthesis and the humoral immunity of animals [54]. Our study revealed a significant decrease in serum creatinine and urea in the Ch/Qu group compared to the Ctrl group. Creatinine and urea are types of non-protein nitrogen related to protein catabolism [55]. The main source of creatinine in serum is associated with the degradation of creatine in animal muscle. Creatinine levels in the blood may increase when diarrhea occurs due to the increased mobilization of muscle protein to compensate for the lack of nutrient absorption. Thus, the decrement of serum creatinine could be associated with the reduction of nutrient availability. Despite the small decrement of digestibility in the Ch/Qu diet observed in the in vitro trial, this scenario can lead to decreased nutrient utilization (in particular, protein availability).

Moreover, we can hypothesize that there was a shift on mild protein metabolism principally associated with the protein binding properties of tannins causing depression of digestive capacity in the small intestine and showed the opposite signs of a growth promoting substance. In fact, in the Ch/Qu group, we also detected a decrement in serum urea, another important indicator of protein status and of feed efficiency [56]. Thus, the decrement of blood urea concentration could be due to the increase of the synthesis of bacterial proteins [57,58]. Tannins promote bacterial growth in the large intestine, which are able to formulate undigested substrate. We found an increased fecal content of polyphenols and nitrogen concentration in the Ch/Qu group, which could be associated with the bio-accessibility and degradation of tannins in the intestinal tract [57,59].

Literature studies showed that proanthocyanidins were not completely absorbed in the gastrointestinal tract, resulting in a higher polyphenol content in the feces of pigs [59]. Additionally, several studies have shown that an increased concentration of fecal polyphenols and nitrogen may be due to the ability of tannins to form stable complexes with proteins [48,59]. In the present study, the positive correlation between phenolic compound intake and fecal N concentration could indicate that phenolic compounds may form protein-tannin complexes and could decrease protein digestibility [48,57,58]. The negative correlation between fecal nitrogen and blood urea could explicate our results. In fact, similar results were reported in the literature where a higher excretion of N through feces was associated with a reduction in blood urea concentration [60].

In light of this, we could hypothesize a possible shift in protein metabolism, which has led to the modulation of serum creatinine, serum urea concentration and fecal metabolites principally related to protein utilization and absorption in the Ch/Qu group.

## 5. Conclusions

Tannins are used mainly for their antimicrobial and antioxidant properties in animal nutrition. In our study, we observed a slight reduction of Ch/Qu diet digestibility and protein utilization, but no effects on feed intake and growth performance were observed. Moreover, in vitro results on IPEC-J2 cells showed that the presence of Ch/Qu tannins in the diet did not impair intestinal cell viability. Furthermore, Ch/Qu supplementation modulated serum creatinine and urea concentration, probably due to a modulation of the entire intestine digestive capacity, which also led to increased fecal nitrogen concentration.

## Figures and Tables

**Table 1:** Ingredient composition of the experimental diets administered to weaned piglets (control (Ctrl), *n* = 60; chestnut/quebracho (Ch/Qu), *n* = 60) from day 0 to day 40 of the experimental trial on an as-fed basis.

Ingredients ^1,2,3,4,5^, as % of Fed Basis	Ctrl	Ch/Qu
Barley meal	25.15	25.00
Wheat meal	19.41	19.07
Corn meal	14.03	13.50
Corn flakes	4.85	4.80
Soybean meal	4.65	4.60
Soybean protein	4.11	4.10
Bakery meal	4.00	4.00
Dextrose monohydrate	3.50	3.50
Wheat middlings	4.32	4.30
Fermented milk product	3.00	3.00
Fish meal	2.50	2.50
Milk whey powder	2.50	2.50
Coconut oil	1.00	1.00
Soy oil	1.00	1.00
Plasma, meal	1.00	1.00
Dicalcium phosphate	0.85	0.80
Animal fats, lard	0.70	0.70
L-Lysine	0.50	0.50
Acidity regulators ^5^	1.00	1.00
Benzoic acid	0.40	0.40
L-Threonine	0.34	0.34
DL-Methionine	0.35	0.35
Sodium chloride	0.26	0.24
Vitamins	0.24	0.24
L-Valine (96.5%)	0.14	0.14
L-Tryptophan	0.08	0.05
Copper sulfate	0.04	0.04
Ch/Qu Tannins ^6^	-	1.25

^1^ Ctrl: basal diet; Ch/Qu: basal diet with tannins (1.25%). ^2^ Nutrient and digestible energy content was calculated using Plurimix software (Fabermatica, CR, Italy). ^3^ Nutrient and digestible energy content (expressed the as-fed basis) of diet: dry matter (DM), 89.37%; crude protein, 16.92%; crude Fat, 5.06%; crude fiber, 3.15%; DE, 3.43 Mc/Kg. DE = digestible energy content estimated from NRC (2012). ^4^ Supplied the following nutrients per kg of diet: 10,000 UI vitamin A, 1000 UI vitamin D3, 100 mg UI vitamin E, 3 mg vitamin B1, 96.3 mg vitamin B2, 5.8 mg vitamin B6, 27 mg vitamin B5, 0.040 mg vitamin B12, 4.8 mg vitamin K3, 0.19 mg biotin, 35 mg niacinamide, 1.4 mg folic acid, 120 mg choline chloride, 70 mg betaine chloride, 108 mg Fe as FeCO_3_, 38.5 mg Mn as MnO_2_, 112 mg Zn as ZnO, 19.3 Cu as CuSO_4_, 0.58 I as Ca(IO_3_)_2_, 0.29 Se as Na_2_SeO_3_. ^5^ Organic Acids: formic acid, sodium formate, sorbic acid, orthophosphoric acid, calcium formate, citric acid, and fumaric acid; ^6^ Commercial chestnut and quebracho tannin extract (Silvafeed Nutri P/ENC for Swine, Silvateam, Italy).

**Table 2:** Chemical analysis of experimental diets fed to piglets from day 0 to day 40 ^1^ of the in vivo trial (from 35- to 75-day-old piglets).

Principal Nutrients	Experimental Diets ^1^		Tannin Additive
	Ctrl	Ch/Qu	Tannins ^2^
Moisture, %	9.54	9.66	6.28
Crude protein, %	17.40	17.72	3.06
Crude fiber, %	3.27	3.45	nd ^3^
Crude fat, %	4.47	4.94	nd ^3^
Ash, %	5.32	5.71	1.29
Phenolic compound, g TAE/kg	0.79 ± 0.03	2.91 ± 0.06	715.05 ± 51.02

^1^ Ctrl: basal diet; Ch/Qu: basal diet with tannins (1.25%). ^2^ Commercial hydrolysable chestnut tannin extract (Silvafeed Nutri P/ENC for Swine, Silvateam, Italy). ^3^ nd = not detectable. g TAE/kg: tannic acid equivalents.

**Table 3:** Growth performance of weaned piglets fed diets with tannins (Ch/Qu, *n* = 60) or without supplementation (Ctrl, *n* = 60) from day 0 to day 40 ^1^ of the in vivo trial (from 35- to 75-day-old piglets).

Growth Performance	Treatments		SEM	*p*-Value		
	Ctrl	Ch/Qu		Treatment	Day	Treatment × Day
Phenolic compound intake, g/d	0.656 ^a^	2.341 ^b^	0.15	<0.01	<0.01	<0.01
ADFI, kg/d	0.623	0.599	0.02	0.258	<0.01	0.241
BW, kg	13.86	13.64	0.64	0.819	<0.01	0.987
ADG, kg/d	0.292	0.284	0.02	0.729	<0.01	0.651
Feed:Gain, d	1.98	2.04	0.07	0.510	0.040	0.140

^a,b^ Indicate differences between treatment groups at *p* < 0.05 within the same day (Trt × Day: *p* < 0.05). Data are shown as least squares means (LSMEANS) and standard errors of the means (SEM). Ctrl: basal diet; Ch/Qu: basal diet with tannins (1.25%). ADFI: average daily feed intake; BW: body weight; ADG: average daily gain.

**Table 4:** The biochemical concentration of serum metabolites in control (Ctrl, *n* = 12) and tannin (Ch/Qu, *n* = 12) groups on day 40 of the in vivo trial (75-day-old piglets) with reference ranges for pigs.

Blood ^1^	Treatments ^2^		SEM	*p*-Value	Reference Range
	Ctrl	Ch/Qu		Treatment	
Total protein content, g/L	52.88	52.18	1.89	0.798	44–74 [41,42,43]
Albumin, g/L	19.30 ^a^	23.05 ^b^	0.88	0.006	19–39 [42]
Globulin, g/L	33.58	29.80	1.55	0.053	19–41 [41,43]
A/G ratio	0.58 ^a^	0.82 ^b^	0.04	0.001	0.50–2.2 [41,42,43]
ALT-GPT, IU/L	38.33	33.82	2.51	0.218	36–117 [43]
AST-GOT, IU/L	54.17	48.09	3.41	0.221	21–98 [42]
ALP, UI/L	165.67	186.00	12.58	0.266	46–341 [43]
Glucose, mmol/L	5.00	5.05	0.25	0.878	3.5–8.6 [41]
Urea, mmol/L	2.18 ^a^	0.95 ^b^	0.19	< 0.001	0.90–8.89 [41,42]
Creatinine, μmol/L	78.92 ^a^	54.82 ^b^	4.18	0.001	67–172 [42]
Total bilirubin, umol/L	1.98	1.67	0.13	0.107	0.9–3.4 [42]
Total cholesterol, mmol/L	2.51	2.38	0.13	0.479	1.3–4.2 [41,42,43]
Triglycerides, mmol/L	0.68	0.58	0.06	0.259	0.3–2.7 [41]
HDL, mmol/L	0.77	0.75	0.03	0.667	-
LDL, mmol/L	1.60	1.52	0.10	0.532	-
Calcium, mmol/L	2.28	2.29	0.06	0.923	2.02–3.21 [42]
Phosphorus, mmol/L	3.05	3.02	0.08	0.774	1.46–3.45 [42]
Magnesium, mmol/L	0.85	0.80	0.02	0.214	0.9–1.2 [41]

^1^ A/G = albumin/globulin; ALT-GPT = alanine aminotransferase; AST-GOT = aspartate aminotransferase; ALP = alkaline phosphatase; HDL = high-density lipoprotein; LDL = low density lipoprotein. ^a,b^ Indicates differences among treatment groups at *p* < 0.05 within the same day. Data are shown as least squares means (LSMEANS) and standard errors of the means (SEM). ^2^ Ctrl: basal diet; Ch/Qu: basal diet with tannins (1.25%).

**Table 5:** Fecal metabolites of weaned piglets fed diets with tannins (Ch/Qu, *n* = 6) or without tannin supplementation (Ctrl, *n* = 6) on day 40 ^1^ of the in vivo trial (from 35- to 75-day-old piglets).

Feces	Treatments ^2^		SEM	*p*-Value
	Ctrl	Ch/Qu		Treatment
Phenolic compound, g TAE/kg	0.34 ^a^	0.50 ^b^	0.05	0.047
Nitrogen, g/kg ^3^	36.02 ^a^	41.35 ^b^	1.00	0.002
Ammonia, g/100 g	2.26	3.20	1.89	0.684
Urea, g/100 g	0.62	0.99	0.23	0.235

^a,b^ Indicates differences among treatment groups at *p* < 0.05 within the same day. ^1^ Data are shown as least squares means (LSMEANS) and standard errors of the means (SEM). ^2^ Ctrl: basal diet; Ch/Qu: basal diet with tannins (1.25%). ^3^ Data are shown as fresh weight. g TAE/kg: tannic acid equivalents.

**Table 6:** Correlation (Spearman correlation) among feed, feces (*n* = 6/treatment) and blood parameters (*n* = 6/treatment) on day 40 ^1,2^ of the in vivo trial (from 35- to 75-day-old piglets).

Feces	Parameter	Spearman r	*p*-Value
Fecal nitrogen, g/kg DM	Blood urea, mmol/L	−0.86	0.014
	Phenolic compound intake, g/d	0.89	0.007

^1^ Ctrl: basal diet; Ch/Qu: basal diet with tannins (1.25%). ^2^ Spearman correlation considering the experimental groups (Ctrl and Ch/Qu).

## Supplementary Materials

The following are available online at https://www.mdpi.com/2076-2615/10/11/1945/s1, Figure S1: The clinical signs of diarrhea of weaned piglets fed diets with tannins (Ch/Qu, *n* = 60) or without (Ctrl, *n* = 60) supplementation from day 0 to day 40 of in vivo trial.
