# Supplementary material for: Evaluation of Dietary Administration of Chestnut and Quebracho Tannins on Growth, Serum Metabolites and Fecal Parameters of Weaned Piglets
Source: Animals (Basel). 2020 Oct 22;10(11):1945. doi: 10.3390/ani10111945 (PMC7690424; doi:10.3390/ani10111945)
Supplement: Supplementary file 1 [file animals-10-01945-s001.pdf]

Figure 1. The clinical signs of diarrhea of weaned piglets fed diets with tannins (Ch/Qu, n=60) or without (Ctrl, n=60) supplementation from day 0 to day 40 of in vivo trial.

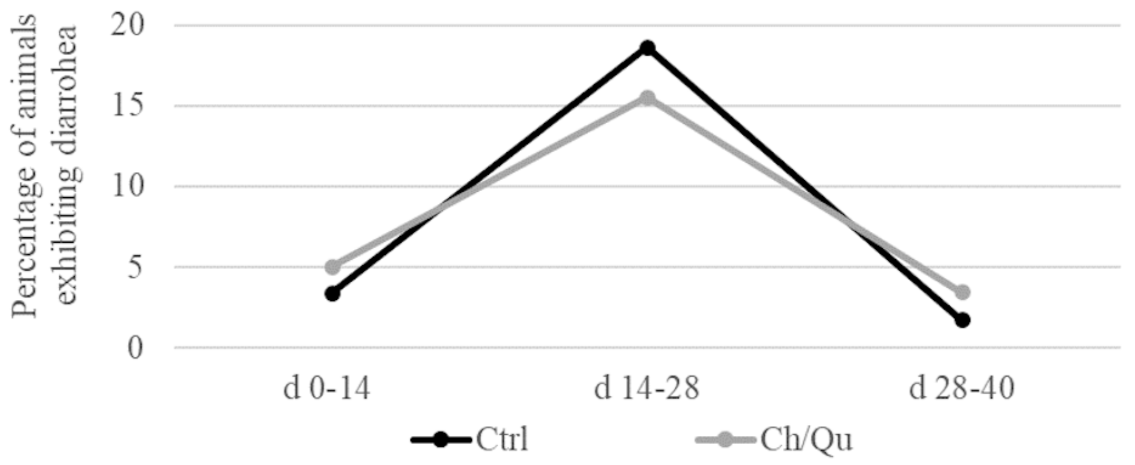

The incidence of diarrhea was calculated based on the number of piglets with clinical sign of diarrhea.
